# Low Hemoglobin Level Is Associated with the Development of Delirium after Hepatectomy for Hepatocellular Carcinoma Patients

**DOI:** 10.1371/journal.pone.0119199

**Published:** 2015-03-13

**Authors:** Yao-Li Chen, Hui-Chuan Lin, Kuo-Hua Lin, Li-Si Lin, Chia-En Hsieh, Chih-Jan Ko, Yu-Ju Hung, Ping-Yi Lin

**Affiliations:** 1 Department of General Surgery, Changhua Christian Hospital, Changhua, Taiwan; 2 Transplant Medicine & Surgery Research Centre, Changhua Christian Hospital, Changhua, Taiwan; 3 Department of Senior Citizen Welfare and Business, Hung Kuang University, Taichung, Taiwan; 4 School of Medicine, Chung Shan Medical University, Taichung, Taiwan; 5 School of Medicine, Kaohsiung Medical University, Kaohsiung, Taiwan; 6 Department of Nursing, Central Taiwan University of Science and Technology, Taichung, Taiwan; Yonsei University College of Medicine, REPUBLIC OF KOREA

## Abstract

**Background:**

Hepatocellular carcinoma (HCC) is one of the most common malignant tumors worldwide and liver resection is the only potential curative treatment option for those patients. Postoperative complications specific to elderly surgical patients such as delirium will be increasingly relevant in the coming decades. Herein, we aimed to investigate the risk factors for postoperative delirium in patients who have received hepatectomy for HCC.

**Methods:**

This is a single medical center observational study and the study subjects comprised 401 individuals who underwent liver resection for hepatocellular carcinoma during January 2009 to October 2013. Multivariate analysis was used to examine whether preoperative, intra-operative, or postoperative variables were associated with the development of delirium.

**Results:**

Of the 401 patients who underwent hepatectomy, 34 developed postoperative delirium (8.4%). In the majority of those patients, symptoms and signs of the syndrome occurred on postoperative day 2 and the mean duration of symptoms was 3.61 ± 3.71 days. Multivariate analysis revealed that advanced age (>71 years) [odds ratio (OR) = 1.133, 95% confidence interval (CI): 1.071–1.200, p<0.001], prolonged operative time (>190 minutes) (OR = 1.009, 95% CI: 1.000–1.017, p = 0.038), a decreased postoperative hemoglobin level (< 10.16 g/dL) (OR = 0.777, 95% CI: 0.613–0.983, p = 0.036), and history of hypnotic drug use (OR = 3.074, 95% CI: 1.045–9.039, p = 0.041) were independent risk factors for the development of postoperative delirium after hepatectomy.

**Conclusions:**

Although the mechanism of postoperative delirium is not well understood, numbers of studies have shown that patients with postoperative delirium tend to have prolonged hospital stay, worse postoperative outcome and an increased risk of short- and long-term mortality. In this study, we found that advanced age, prolonged operative time, postoperative low hemoglobin level and history of hypnotic drug use are independent risk factors for postoperative delirium.

## Introduction

Delirium is an etiologically non-specific organic cerebral syndrome characterized by concurrent disturbances of consciousness and attention, perception, thinking, memory, psychomotor behavior, emotion and the sleep–wake schedule. The duration is variable and the degree of severity ranges from mild to very severe **[1]**.

Postoperative delirium typically develops 1–4 days after an operation and results in longer hospital stay, additional nursing care, and greater medical costs [**2**]. Studies have also shown that postoperative delirium is associated with an increased risk of mortality [**3**]. Although the mechanisms underlying the development of postoperative delirium are not well understood, the most significant risk factors that have been shown to be involved in the process are age, cognitive impairment, coexisting medical conditions, and psychotropic medication use [**2,3**].

Hepatocellular carcinoma (HCC) is one of the most common malignant tumors worldwide and liver resection is the only potential curative treatment option for those patients. It has been reported that patients with chronic liver diseases or impaired liver function are at increased risk for developing delirium after a surgical procedure **[4**]. However, the risk factors for developing delirium after hepatectomy for HCC patients have not been well investigated. Yoshimura et al. [**5**] once revealed that advanced age, impaired liver function, and advanced cancer stage affect the development of postoperative delirium after liver resection for HCC; yet, the case number enrolled was small. Thus, we conducted a large-scale study to investigate preoperative, intraoperative, and postoperative factors associated with the development of delirium after hepatectomy for HCC patients.

## Materials and Methods

### Patients

This was a retrospective study. All clinical data were obtained through chart review. During the period January 2009 to October 2013, a total of 406 patients without preoperative or postoperative hepatic encephalopathy and received elective liver resection for HCC at our institution was included. The diagnosis of hepatic encephalopathy was based on clinical criteria, and the severity of hepatic encephalopathy was assessed using West Haven Criteria **[6**] for grading of mental status. Biochemical data such as ammonia levels were determined as additional diagnostic variables. Of those patients, 5 required treatment in the intensive care unit (ICU) and were excluded from the study cohort to minimize the effect of post-intensive care syndrome on the organic nature of the development of postoperative delirium. The number of patient in the study, therefore, comprised 401 patients (298 men and 103 women) ranging in age from 26 to 89 years. The patients were divided into two groups based on whether they developed postoperative delirium (n = 34) or did not (n = 367). The study was approved by the institutional review board of the Changhua Christian Hospital and a waiver of patient consent was obtained due to the retrospective nature of the evaluation.

### Diagnosis of Postoperative Delirium

We used the Confusion Assessment Method (CAM) [**7**] to diagnose postoperative delirium. CAM is a widely used diagnostic algorithm for identification of delirium and has been shown to have a high sensitivity (86%) and high specificity (93%) **[8**]. Delirium was diagnosed in patients who exhibited the following features: (1) acute onset of cognitive impairment with a fluctuating course and (2) at least two of the following: (a) perceptual disturbance, (b) disorganized thought, (c) disorientation and memory impairment, (d) altered level of consciousness, or (e) hyperactive or hypoactive psychomotor activity. Abnormal behavior included agitation, frequent summoning of nurses, removal of intra-venous lines, drainage tubes, or dressings; and wandering.

### Risk Factors for Delirium

The variables investigated in this study were similar to those reported in previous studies, namely age, gender, body mass index, diabetes mellitus, hypertension, respiratory disorder, personal history (alcohol, smoking and hypnotic drug use), preoperative and postoperative laboratory test results, Child-Pugh score, operative method (major hepatectomy, defined as resection of ≥ 3 segments. or minor hepatectomy), duration of operation, intraoperative blood loss, postoperative infection events, and ward environment (single room and four-bed room) [**9–15**]. The degree of liver fibrosis was assessed using the METAVIR scoring system (F0 = no fibrosis; F1 = portal fibrosis without septa; F2 = portal fibrosis with rare septa; F3 = numerous septa without cirrhosis; and F4 = liver cirrhosis) **[16**]. Infection included pneumonia, bacteremia, urinary tract infection, intra-abdominal infection, biliary infection and wound infection. History of alcohol, smoking and hypnotic drug use was defined as consumption of these substances for more than six months.

### Statistical Analysis

Continuous variables are presented as mean ± standard deviation (SD) and categorical variables are presented as percentages. The independent t-test was used to determine differences in the means of continuous variables. Categorical variables were compared using the χ^2^ test or Fisher’s exact test when appropriate. Univariate logistic regression was used to assess the significance of risk factors and to obtain odds ratios. Significant predictors in the univariate analyses were then included in a forward, stepwise multivariate logistic regression model to identify the most important risk factors for postoperative delirium. A P-value < 0.05 was considered to represent statistical significance. All statistical analyses were performed using the statistical package SPSS for Windows (Version 17.0, SPSS Inc; Chicago, IL, USA).

## Results

A total of 34 patients out of 401 developed postoperative delirium; the incidence was 8.4%. In the majority of those who developed postoperative delirium, symptoms and signs of the syndrome occurred on postoperative day 2 with a mean duration of 3.61 ± 3.708 days (Table 1).

**Table 1:** Onset and duration of postoperative delirium.

	Delirium	
	n = 34(%)	
Day 1	3(8.8)	
Day 2	11(32.4)	
Day 3	5(14.7)	
Day 4	9(26.4)	
Day 5	6(17.6)	
	Mean±SD	Range
Onset (Postoperative day)	3.12±1.297	1–5
Duration (days)	3.61±3.708	1–21

Table 2 illustrates the demographic and clinical pathological features and the perioperative and postoperative features of the two groups are presented in Table 3. The mean hospital stay was 9.18 ± 3.145 days for patients who did not develop delirium and 13.32 ± 8.608 days for those presented with postoperative delirium (p = 0.026).

**Table 2:** Characteristics of patients in the delirium and non-delirium groups.

	Non-Delirium	Delirium	
Characteristics^a^	n = 367	n = 34	*p*-value
Age	61.23±11.228	71.24±7.447	<0.001
BMI	24.66±3.562	23.02±3.384	0.010
MELD sore	8.24±2.439	9.50±4.548	0.121
Pre-OP ALT (U/L)	62.01±192.203	48.85±54.974	0.696
Pre-OP TB (mg/dL)	0.82±0.384	0.87±0.493	0.459
Pre-OP platelet (10^3^/μl)	173.91±88.456	189.26±98.187	0.338
Pre-OP albumin (g/dL)	3.92±0.478	3.64±0.464	0.001
Creatinine (mg/dL)	1.02±0.666	1.46±1.637	0.133
	(%)	(%)	
Male^b^	277(75.5)	21(76.5)	0.897
Smoking	108(29.4)	11(32.4)	0.721
Alcohol Drinking	59(16.1)	3(8.8)	0.329
Diabetes mellitus	80(21.8)	10(29.4)	0.309
Hypertension	129(35.1)	14(41.2)	0.483
Heart Disease	19(5.2)	4(11.8)	0.120
Lung Disease	15(4.4)	5(14.7)	0.010
Child Pugh classification			0.173
A	360(98.1)	32(94.1)	
B	7(1.9)	2(5.9)	
Fibrosis score			0.396
F0	10(2.8)	0(0)	
F1	72(20.4)	9(27.3)	
F2	95(26.9)	10(30.3)	
F3	74(21.0)	3(9.1)	
F4	102(28.9)	11(33.3)	
Hypnotics	33(9.0)	9(26.5)	0.002

^a^ Data are shown as mean±standard deviation and were compared using the independent T test.

^b^ Data are shown as n (%) and were compared using the chi-square or fisher’s exact test.

BMI, Body Mass Index; MELD score, the model of end-stage liver disease score; Pre-OP, pre-operative; ALT, Alanine aminotransferase; TB, Total bilirubin; Smoking and alcohol drinking: consumption of these substances for more than six months.

**Table 3:** Perioperative and postoperative features of patients in the delirium and non-delirium groups.

	Non- Delirium	Delirium	
Parameters^a^	N = 367	N = 34	*p*-value
Operative time (min)	136.04±47.225	190.91±153.027	0.045
Intraoperative blood loss (ml)	357.78±465.332	758.24±1391.469	0.104
Postoperative peak ALT (U/L)	178.32±206.666	206.28±287.294	0.501
Postoperative peak TB (mg/dL)	2.03±7.185	2.53±2.639	0.719
Postoperative platelet (10^3^/μl)	137.30±58.330	133.84±61.062	0.750
Postoperative albumin (g/dL)	3.09±0.495	2.80±0.520	0.345
Postoperative Na (mmol/L)	135.77±10.284	135.79±4.498	0.990
Postoperative hemoglobin (g/dL)	11.84±1.723	10.16±2.682	<0.001
Postoperative hospital stay	9.18±3.145	13.32±8.608	0.026
	(%)	(%)	
≥ 3 segmental hepatectomy^b^	122(33.2)	18(52.9)	0.021
Infection complication	17(4.6)	7(20.6)	<0.001
Four-bed hospital room	167(45.5)	18(52.9)	0.405

^a^ Data are shown as mean±standard deviation and were compared using the independent T test.

^b^ Data are shown as n (%) and were compared using the chi-square or fisher’s exact test.

ALT: Alanine aminotransferase; TB: Total bilirubin.

In patients with postoperative delirium, most of them had good liver function according to the Child Pugh classification (Child Pugh A: 94.1%; B: 5.9%). Regarding the degree of liver fibrosis, there was no significant difference between the two groups (Table 2).

According to the univariate analysis, advanced age (> 71 years old), low BMI index (< 23.02 kg/m^2^), low albumin level before operation (< 3.64 g/dL), lung disease comorbidity, history of hypnotic drug use, prolonged operative time (> 190 minutes), major liver resection (≥ 3 segments), postoperative hemoglobin level, and postoperative infection complications were factors associated with the development of postoperative delirium (Table 4).

**Table 4:** Risk factors for postoperative delirium by univariate analysis and multivariate analysis.

	Univariate analysis			Multivariate analysis		
Parameters	Odds ratio	95% CI	*p*	Odds ratio	95% CI	*p*
Age	1.120	1.069–1.174	< 0.001	1.133	1.071–1.200	< 0.001
BMI	0.866	0.775–0.967	0.011	-	-	-
Preoperative albumin (g/dL)	0.340	0.174–0.665	0.002	-	-	-
Operative time (min)	1.008	1.004–1.013	0.001	1.009	1.000–1.017	0.038
Postoperative hemoglobin (g/dL)	0.629	0.510–0.775	< 0.001	0.777	0.613–0.983	0.036
Lung disease	3.782	1.293–11.062	0.015	-	-	-
Hypnotic drug use	3.622	1.561–8.404	0.003	3.074	1.045–9.039	0.041
≥ 3 segmental hepatectomy	2.259	1.113–4.584	0.024	-	-	-
Infection complication	5.338	2.037–13.987	0.001	-	-	-

BMI: Body Mass Index.

Multivariate analysis revealed that advanced age (OR = 1.133; 95% CI: 1.071–1.200), prolonged operative time (OR = 1.009; 95% CI: 1.000–1.017), a decreased postoperative hemoglobin level (OR = 0.777; 95% CI: 0.613–0.983), and history of hypnotic drug use (OR = 3.074; 95% CI: 1.045–9.039) were independent risk factors for the development of postoperative delirium after hepatectomy (Table 4).

Patients who developed delirium after hepatectomy were significantly older than those who did not show signs of delirium (71.24 ± 7.447 vs. 61.23 ± 11.228, p<0.001) and had a longer operative time (190.91 ± 153.027 vs. 136.04 ± 47.225, p = 0.045) than patients who did not develop the syndrome. Furthermore, patients who developed delirium had a significantly lower mean postoperative hemoglobin level than patients who did not show symptoms or signs of the syndrome (10.16 ± 2.682 vs. 11.84 ± 1.723, p<0.001).

## Discussion

Advanced age, impaired liver function, and advanced cancer stage were independent risk factors for postoperative delirium after hepatectomy for HCC was once discovered by Yoshimura et al **[5**] and the incidence of post-operative delirium was 17%. In our study, the incidence decreased to 8.4% and besides advanced age; we found that a history of hypnotic drug use, prolonged operation time, and low postoperative hemoglobin concentration were also independent risk factors for the development of delirium after hepatectomy.

A number of studies have shown that patients of advanced age are prone to develop delirium after operations mainly due to cholinergic deficiency [**17**] and dopaminergic excess [**18**]. Acetylcholine may play a role in many of the symptoms of delirium. Excess anticholinergic activity is associated with behavioural inhibition, whereas anticholinergics are associated with hyperactivity. Elderly patients are more vulnerable to anticholinergic effects as the functionality of cholinergic transmission decreaseswith age. Dopamine is an neurotransmitter for motor function, attention and cognition; which is important in the genesis of delirium.

There are five types of dopamine receptors in human, activation of D1 and D5 receptors increases secretion of acetylcholine, whereas D2, D3 and D4 activation might decrease acetylcholine secretion. D1 and D2 receptors decrease with age, which could increase the likelihood of delirium in elderly patients. Though the pathophysiology of postoperative delirium is not well understood, those are indirect evidence why the elderly are prone to develop postoperative delirium.

O'Keeffe et al found that drug-related side effects may contribute to postoperative delirium in the elderly [**19**]. In a systematic review article, Steiner [**14**] reported that use of hypnotic agents such as benzodiazepines, opioids and anticholinergic agents are precipitating factors for the development of postoperative delirium. Guidelines published by the National Clinical Guideline Center [**20**] also mentioned that many pharmacological agents, such as benzodiazepines, antipsychotics, anticholinergic, H2-receptor antagonists, mood stabilizing drugs, non-steroidal anti-inflammatory drugs, and opioids are risk factors for delirium. In our institution, benzodiazepines such as Alprazolam, Lorazepam and Zolpidem are the most common hypnotic agents prescribed for insomnia; this might explain why patients who took hypnotic agents were at risk of developing postoperative delirium. Moreover, Benzodiazepines and opioids are frequently administered during operations for sedation and analgesia. We thought this might be the reason that patients with long operation time tended to experience postoperative delirium.

Yoshimura et al [**5**] found that decreased albumin concentration after hepatectomy was a risk factor for postoperative delirium. Although this was not observed in our study, we did find that patients with hypoalbuminemia before operation were at greater risk of developing postoperative delirium. This condition was also observed by Robinson et al [**21**]. Whether hypoalbuminemia is a consequence of liver injury caused by HCC or liver dysfunction due to aging is not clear; however, low serum albumin level has been shown to affect the metabolism of drugs and to be associated with postoperative delirium [**22–24**].

In our study, low postoperative hemoglobin level was associated with the incidence of postoperative delirium after hepatectomy for HCC. This issue was not mentioned in Yoshimura’s study and was first discovered to be risk factors for delirium in HCC patients. Older patients tend to have respiratory comorbidities such as hypoxia and hypercapnia, which reduce cerebral circulation flow and may lead to postoperation delirium. Indeed, in our study, the proportion of patients with respiratory comorbidities was higher in the delirious group than in the non-delirious group. Furthermore, intraoperative blood loss is hard to be avoided in liver resection operation due to liver dysfunction with cirrhosis change and coagulopathy. As a result, we adjust the time interval and frequency of the Pringle maneuver during the operation to reduce liver injury. In this study, we found that low postoperative hemoglobin level was an independent risk factor for delirium. Although a similar finding has been reported in other studies [**25, 26**], those studies involved small sample sizes. To the best of our knowledge, our study is the first large-scale study to reveal that low postoperative hemoglobin level is a risk factor for delirium after hepatectomy for HCC. We suggest that maintaining hemoglobin levels > 11 g/dL might help reduce the risk of postoperative delirium.

In this study, we also investigated whether room occupancy in hospital wards had an impact on the development of postoperative delirium. In Taiwan, the National Health Insurance program covers the cost of hospital stay as long as a patient stays in a four-bed occupancy hospital room. We thought that four patients with care assistants in the same space might influence each other especially, sleep rhythm. As a result, we discovered that there was no significant difference in the proportion of patients who stayed in four-bed rooms between the delirium group (18 of 34, 52.9%) and the non-delirium group (167 of 367, 45.5%) (p = 0.405).

## Conclusion

Although the mechanism of postoperative delirium is not well understood, numbers of studies have shown that patients with postoperative delirium tend to have prolonged hospital stay, worse postoperative outcome and an increased risk of short- and long-term mortality. In this study, we found that advanced age, prolonged operative time, postoperative low hemoglobin level and history of hypnotic drug use are independent risk factors for postoperative delirium.
